# Effectiveness and equity impacts of traffic restriction schemes outside schools: a controlled natural experimental study

**DOI:** 10.1186/s12966-025-01858-w

**Published:** 2025-12-16

**Authors:** Richard Patterson, Emma Grace Carey, Kate Garrott, Yuru Huang, David Ogilvie, Sophie Hadfield-Hill, Andy Cope, Adrian Davis, Esther van Sluijs, Jenna Panter

**Affiliations:** 1 MRC Epidemiology Unit, School of Clinical Medicine, University of Cambridge, Cambridge, UK; 2 School of Health Sciences, University of Birmingham, Birmingham, UK; 3 Department of Human Ecology, University of California, Davis, UK; 4 School of Geography, Earth and Environmental Sciences, University of Birmingham, Birmingham, UK; 5 Walk Wheel Cycle Trust, Newcastle Upon Tyne, UK; 6 Edinburgh Napier University, Edinburgh, UK

**Keywords:** Walking, Cycling, School travel, Evaluation, Active commuting

## Abstract

**Background:**

Active travel (such as walking, cycling and scooting) has a range of benefits for human and planetary health, whereas driving children to school contributes substantially to motor vehicle traffic at peak times. Local governments have collaborated with schools to implement traffic restriction schemes, in which motor vehicle access around schools is restricted at drop-off and pick-up times. We examined the impacts of these schemes on how children travel to school, and how these differed between socio-economic groups, in England and Scotland.

**Methods:**

In this controlled before-and-after natural experimental study, we used data collected by primary schools on children’s mode of travel to school between 2012 and 2023. We matched each intervention school to two control schools based on area-level deprivation, urban–rural status, school size, baseline prevalence of active travel to school, and geographical region. We used fixed-effects regression models to conduct difference-in-difference analyses of the percentage of pupils using active modes of transport and private motor vehicles, adjusting for potential confounding factors. We examined absolute and relative differences and differential effects by stratifying analyses by geographical region, method of enforcement, area-level deprivation, and urban–rural status.

**Results:**

We used data from 498 schools (166 intervention and 332 control) at which on average 70% of children travelled to school by active modes at baseline, with no significant difference between intervention and selected control schools (*p* = 0.79). The proportion of pupils in intervention schools travelling by active modes increased by 5.9 absolute percentage points (95% CI: 2.5 to 9.1), and the proportion travelling by private motor vehicle decreased by 5.3 points (2.5 to 8.2), relative to control schools. The results for relative changes were similar, the patterns were consistent between jurisdictions and no differences were seen in other stratified analyses.

**Conclusion:**

We found that after primary schools implemented schemes, a greater proportion of children walked, cycled or scooted to school and a smaller proportion were driven. These findings suggest that wider roll-out of these schemes might contribute to promoting active travel in children, and perhaps, to improving health. Improving the availability, quality and consistency of routinely collected data on travel to school would facilitate future research into these schemes.

**Supplementary Information:**

The online version contains supplementary material available at 10.1186/s12966-025-01858-w.

## Background

Levels of physical activity in many countries are low and it is known that physical activity is beneficial for health. The journey to school is an important opportunity to integrate physical activity into children’s daily routines. Children who walk or cycle to school engage in more physical activity overall than those who are driven [1], which can be expected to result in environmental and health gains and reduce healthcare costs [2–4]. In addition, using motor vehicles can be harmful to health, whether by causing road traffic injuries or by contributing to air and noise pollution and physical inactivity [5]. These harms may be concentrated around schools, where streets may become heavily congested with vehicles, pedestrians and cyclists at the beginning and end of the school day. Children are particularly vulnerable to the effects of atmospheric pollutants, due to their higher ventilation rates and developing immune, neurological and respiratory systems [6, 7].

Most studies of interventions to promote active travel in children have focused on those implemented by schools [8, 9]. While there is some evidence that school-based interventions can be effective [8–10], the literature is dominated by uncontrolled, unrepresentative studies [11, 12] and the findings are heterogeneous, suggesting that intervention design and contextual factors may be important determinants of effectiveness [11]. Most studies have evaluated educational, informational or social support interventions such as walking buses, school travel planning and cycle training. Fewer have evaluated environmental or policy interventions such as improving cycle lanes and crossings [9] or charging drivers to enter particular zones [13], and for the population as a whole there is more evidence about the effects of positive incentives to walk or cycle than about the effects of disincentives to drive [14].

Making physical changes to the built environment to discourage the use of motor vehicles is an approach that has been shown to reduce car use, encourage walking and cycling and can help to reduce health inequities [15]. This can be done in various ways including removing parking spaces, narrowing road lanes, replacing roads with walking or cycling infrastructure, or installing low-traffic neighbourhoods – schemes that filter motor traffic away from residential streets, while continuing to allow walking and cycling. Evidence shows that these can reduce motor traffic [16, 17], and increase physical activity [18, 19], although the evidence base is heterogeneous and conclusions are difficult to draw suggesting that the impacts of these interventions should be explored further.

Traffic restriction schemes outside schools, also known as ‘school streets’ or ‘no car zones’, are a related type of intervention intended to discourage motor vehicle use during school drop-off and pick-up times. These may be implemented in different places for different reasons, for example to improve road safety, reduce air pollution or increase active travel. School streets originated in Bolzano, Italy in the 1980s. An early UK example was introduced by East Lothian Council in 2014 [20] and schemes have proliferated in the UK in recent years, following increased policy interest, guidance on implementation [20] and investment [21]. These schemes remain largely unevaluated in spite of their growing popularity and perceived effectiveness, although a review of grey literature based on uncontrolled studies using small samples found evidence that they reduced motor vehicle use and increased active travel, and were supported by caregivers [22]. In the last year two small scale qualitative studies of schemes have been published from a small town in Eastern England and in Newcastle, UK [23, 24] but quantitative studies are lacking.

In this controlled natural experimental study, we assessed the effects of traffic restriction schemes on children’s levels of active travel to school and explored differential impacts by geographical region (Scotland, London, and the rest of England), enforcement method and context of the school location.

## Methods

### Study design

We used a difference-in-difference approach to estimate the impact of traffic restriction schemes on changes in travel behaviour, incorporating data from control schools who did not receive the intervention. We use routinely collected longitudinal data analysed at the school-level from England and Scotland.

### Intervention

Traffic restriction schemes place temporary restrictions on motor vehicles outside schools at the beginning and end of the school day. They encourage people travelling by motor vehicle to park further from school and complete the rest of the journey on foot (‘park-and-stride’) or by bike, or to leave their car at home. Exemptions vary between local governments, but often include local residents, people with disabilities, delivery drivers and emergency services. Even if exempt, local residents are encouraged to avoid travel and limit visitors during these times.

Schemes take a variety of forms across the UK, with differing approaches to enforcement. Some local authorities rely on signage, with or without cameras. Others are overseen by volunteers who put up physical barriers such as bollards, planters or ropes to close the streets. The estimated costs of implementing Schol Streets vary from £5,000 to £10,000 (one-off costs) which includes legal fees and physical measures (equivalent to around US $6,000 to $12,000) per school, and can be higher depending on the enforcement methods used [20].

### Data

#### Exposure: Intervention schools

Travel for Life (part of Transport for London) and Walk Wheel Cycle Trust (formerly Sustrans), a charity that advocates for active travel and works with local governments to deliver schemes, both provided a list of schools with traffic restriction schemes (including operational permanent schemes, pilot schemes, one-day interventions, or schemes planned but not yet implemented). The research team also conducted a web search to identify other schools outside London that had implemented schemes but had not involved Walk Wheel Cycle Trust. Our final list of intervention schools included only state primary schools (publicly funded schools for children aged 4 to 11 (in England) and to 12 (in Scotland) with operational permanent traffic restriction schemes. We excluded independent and specialist schools, whose catchment areas are generally substantially larger than those of state primary schools. We also excluded schools operating across more than one site, because data about travel patterns (see below) were not site-specific.

#### Outcome: Children’s travel patterns

We explored the suitability of national data sources on children’s travel patterns (Supplementary Material, Table S1) and decided that hands-up survey data collected by schools would be most suitable due to their national coverage and homogeneous method of assessment. Hands-up surveys are completed in class by teachers asking children “How do you normally travel to school?” Children answer by raising their hand when their response option is called, and staff count the number of hands raised for each option. In principle, schools should note the number of pupils present on a given day and report data from at least 80% of children. Some, but not all, schools complete the survey more than once a year. Guidance is available [25] and hands-up surveys have been shown to be valid and reliable in primary school children [26]; those authors found test–retest reliability κ = 0.96 and criterion validity κ = 0.91 but levels of adherence in the datasets we used are unknown.

We accessed hands-up survey data through Modeshift (a national organisation that promotes sustainable travel) for primary schools in England outside London, through Transport for London for those in London, and through the Walk Wheel Cycle Trust for those in Scotland. We were unable to obtain any before-and-after data from schools in Wales. Data sharing agreements were arranged according to the requirements of each data provider.

We aggregated and recoded the reported modes of transport to allow for response categories to be harmonised between region/nation, resulting in a sixfold classification: walking/buggy, cycling/scooting/skating, and park-and-stride (together defined as ‘active’ modes) and public transport, private motor transport and other (Supplementary Material, Table S2). We excluded other modes from the analysis as very small numbers of pupils reported using them. We included park-and-stride as an active mode because it explicitly includes some walking, cycling or scooting, and because measures to support this (e.g. an official park-and-stride parking point) are considered part of the schemes at some schools [21]. We computed percentage mode share by dividing the number of students travelling by each mode of transport by the total number of students and multiplying by 100.

#### Data on schools

Each school’s Unique Reference Number was used to link to data on the number of pupils on roll and confirm its postal address, which was used to link to Index of Multiple Deprivation (IMD), a measure of area-level deprivation and urban rural status [27, 28]. Schools in Scotland are assigned a Scottish Exchange of Educational Data code, which we used in the same way. Further details on school covariates are given in Supplementary Material, Table S3.

## Potential sample

### Time window selection

Time windows were selected based on the availability of data from intervention schools, with the goal of maximising the number included. We tested all combinations of window sizes, ranging from one to three years both before and after intervention resulting in nine possible combinations. A window of three years pre- and one year post-intervention maximised the sample size of available schools and was used for the main analysis. The three-year pre-intervention window conferred the additional benefit of allowing us to assess pre-intervention trends as required for the parallel trends assumption in difference-in-differences analysis. A longer post-intervention window may have allowed us to observe longer-term behaviour changes, but due to the recency of implementation of most schemes and the delay between data collection and availability, this would have resulted in a considerably smaller sample size.

### Control schools

We matched intervention schools with control schools, which were selected from among all available state primary schools with no intervention (see above). Due to the limited number of controls available within some local authorities, we did not force matching within a given local authority. Schools were matched based on geographical region (London, England excluding London, and Scotland), baseline active travel (z-score of percentage of students travelling to school by active modes in the pre-intervention year), IMD decile, school size (z-score of number of pupils on roll) and urban–rural status (urban vs. rural).

We matched using a 2 (controls):1 (intervention) matching method resulting in 322 control schools. We used Mahalonobis distance with nearest neighbour matching methods) as this ratio had the lowest mean absolute standard bias, conducting the matching separately for each year of intervention as the pool of potential control areas differed because of data availability. Both Mahalanobis and propensity score matching are statistical methods used to find controls which are similar to treated units on a number of covariates. Propensity score matching does this by creating a score based on *all covariates on average* which represents a unit’s likelihood of receiving a treatment, and matching on this score [29]. In contrast, Mahalanobis distance matching finds controls which are most similar to intervention units on *each* of the covariates [30, 31]. We compared results for propensity score matching and Mahalanobis distance matching, and chose Mahalonobis distance as it resulted in the best covariate balance (Supplementary Material, Fig. 1 and 2).

We assessed pre-intervention parallel trends visually in both control and intervention groups. We compared all potential control and intervention schools, as well as those used in the analysis, on a range of baseline characteristics including all those used for matching, and tested differences using unpaired t-tests for continuous measures and chi-squared tests for categorical measures (Table 1).

**Table 1:** School characteristics at baseline^*^

	A: All control schools	B: Selected control schools	C: All interventionschools	D: Selected intervention schools	Difference between A and D (*p*-value**)	Difference between B and D (*p*-value)***	Difference between C and D (*p*-value)
Number of schools	1891	332	572	166			
Baseline active travel*							
Mean % (SD)	60.3 (18.0)	70.0 (12.0)	n/a	70.3 (12.4)	< 0.001 < 0.001	0.770	n/a
Baseline motor travel*							
Mean % (SD)	33.3 (16.7)	22.2 (10.5)	n/a	21.8 (11.1)	< 0.001	0.730	n/a
Baseline public transport*							
Mean % (SD)	6.1 (9.4)	7.6 (6.5)	n/a	7.4 (6.5)	0.026	0.761	n/a
School size							
Number on roll (SD)	339.3 (176.1)	424.3 (169.1)	414.4 (186.9)	428.6 (166.7)	< 0.001	0.783	0.348
Camera enforcement							
No	n/a	n/a	266 (46.5%)	67 (40.4%)	< 0.001	n/a	0.190
Yes	n/a	n/a	306 (53.5%)	99 (59.6%)			
Index of multiple deprivation quintile							
1—Most deprived	338 (17.9%)	61 (18.4%)	139 (24.3%)	38 (22.9%)	0.001	0.294	0.357
2	394 (20.8%)	108 (32.5%)	184 (32.2%)	44 (26.5%)			
3	348 (18.4%)	70 (21.1%)	116 (20.3%)	40 (24.1%)			
4	412 (21.8%)	50 (15.1%)	68 (11.9%)	18 (10.8%)			
5—Least deprived	399 (21.1%)	43 (13.0%)	65 (11.4%)	26 (15.7%)			
Geographical region							
England excl. London	996 (52.7%)	12 (3.6%)	75 (13.1%)	6 (3.6%)	< 0.001	n/a	< 0.001
London	700 (37.0%)	250 (75.3%)	430 (75.2%)	125 (75.3%)			
Scotland	195 (10.3%)	70 (21.1%)	67 (11.7%)	35 (21.1%)			
Urban/rural status							
Rural	270 (14.3%)	8 (2.4%)	5 (0.9%)	4 (2.4%)	< 0.001	n/a	0.236
Urban	1621 (85.7%)	324 (97.6%)	567 (99.1%)	162 (97.6%)			

^*^for intervention schools and matched controls, “baseline” was considered as 3 years prior to the intervention implementation (ranging from 2012 to 2019). For the sample including all potential controls, covariates were based on the first year for which data was available from 2012 onwards

^**^p-values from chi-square contingency test for categorical variables and an independent sample t-test for continuous variables

n/a Percentages are identical, so p value is 1 (in the case of geographical region matching was forced based on this)

## Sample for analysis

### Intervention sample

**Fig. 1:**
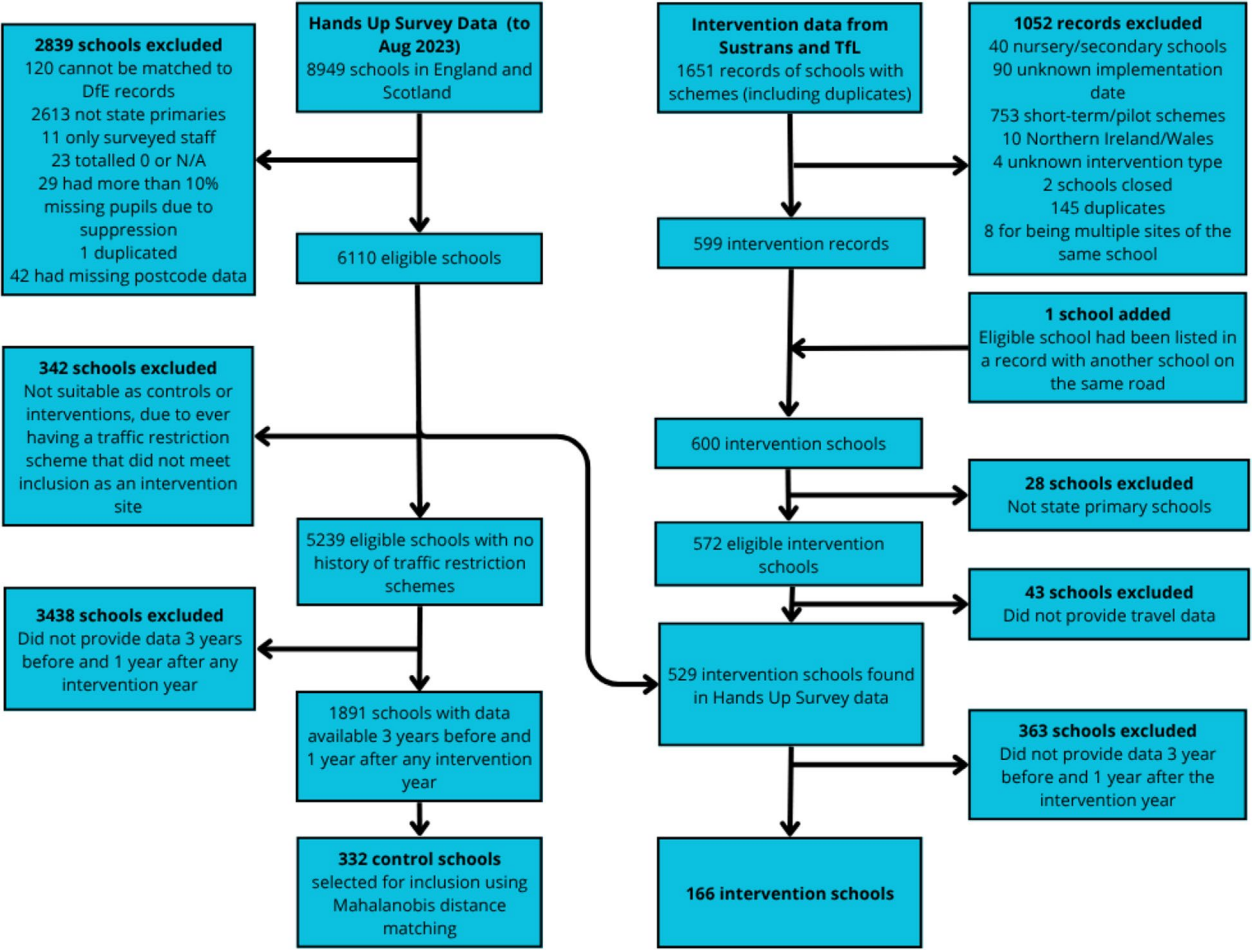
Flowchart showing schools included in the analysis

After collating data sources about traffic restriction schemes, we had a total of 1651 schools (including duplicates). We excluded 1079 schools, leaving 572 eligible state primary schools, of which 166 schools had data for three years pre-implementation and one year post-implementation (Fig. 1). 32 local governments were represented and the numbers of intervention schools per year ranged from three in 2015 to 96 in 2020 (Supplementary Material, Table S4).

### Control sample

We had survey data from 8949 schools: 2999 schools in London, 5548 in the rest of England and 402 in Scotland. We excluded schools which were not state primaries, provided insufficient data or could not be linked with national school data, leaving 6110 potential control schools. We then excluded schools with a traffic restriction scheme of any kind, leaving 5239 potential control schools. This group was then further restricted to 1891 schools that provided travel data matching the chosen time window and for which all covariate data were available for matching. Further information on the control and intervention schools available by year are shown in Supplementary Material, Table S5.

## Analysis

### Statistical analysis

We used a linear two-way fixed-effects model, with school and time fixed effects, to estimate mean differences in the percentage of pupils using travel modes, including an observation for each school for each time point (pre- and post-intervention). We adjusted for number of children on roll (standardised to z-scores), urban/rural status, and index of multiple deprivation (IMD). Intervention effectiveness was estimated as the interaction between time (pre/post intervention) and treatment group (intervention/control) in an individual-level difference-in-difference approach [32]. This represents the difference between intervention and control schools in the change in the percentage of pupils using a given mode of travel before and after the intervention. We computed both the absolute and relative change in these percentages. For example, where the mean baseline prevalence of active travel at intervention schools is 70%, a difference-in-difference estimate of 7% represents an absolute increase of seven percentage points (from 70 to 77%) in the proportion of children using active travel after adjustment for any change observed in the control schools. In this instance, the relative (or proportional) increase in the prevalence of active travel would be 10%.

We also stratified the analysis by region/nation (Scotland, London, and the rest of England) and fitted interactions by method of enforcement (camera monitoring v other) and quintile of area-level deprivation to test for any differences. It was not possible to test for differences by urban–rural status owing to the small number of rural schools. All statistical analyses were performed using R version 4.3.0 [33]. Missing data were not imputed.

### Sensitivity analysis

We performed three sensitivity analyses. First, as linear regression models do not account for the bounded nature of our percentage outcomes, we tested the sensitivity of our results to this consideration using fractional regression models with a logit function. Second, we tested the impact of the time window chosen by repeating the analyses using data from only one year before and one year after the intervention, which maximised the sample size but did not allow comparison of pre-intervention trends. Third, to acknowledge potential differences between intervention and control schools in their enthusiasm for promoting active travel, we repeated the analysis with additional matching on their accreditation status with Modeshift STARS, a national scheme for organisations demonstrating excellence in supporting sustainable travel. This information was only available for schools in England, so this sensitivity analysis was limited to those schools.

## Results

### Comparability of intervention and control schools

Our intervention and matched control schools were similar across several important measurable factors such as baseline (three years prior to intervention) prevalence of active travel, IMD quintile, school size, geographical region and urban–rural status (Table 1). Comparing intervention schools included in this analysis with all intervention schools, there were no significant differences except that a lower proportion of schools outside London were included (*p* < 0.001), mainly because of a lack of data. Intervention schools included in this analysis were more likely to be in more deprived areas, to be in urban areas and to have a higher level of active travel at baseline than all potential control schools (all p < 0.001).

### Changes in travel modes

**Fig. 2:**
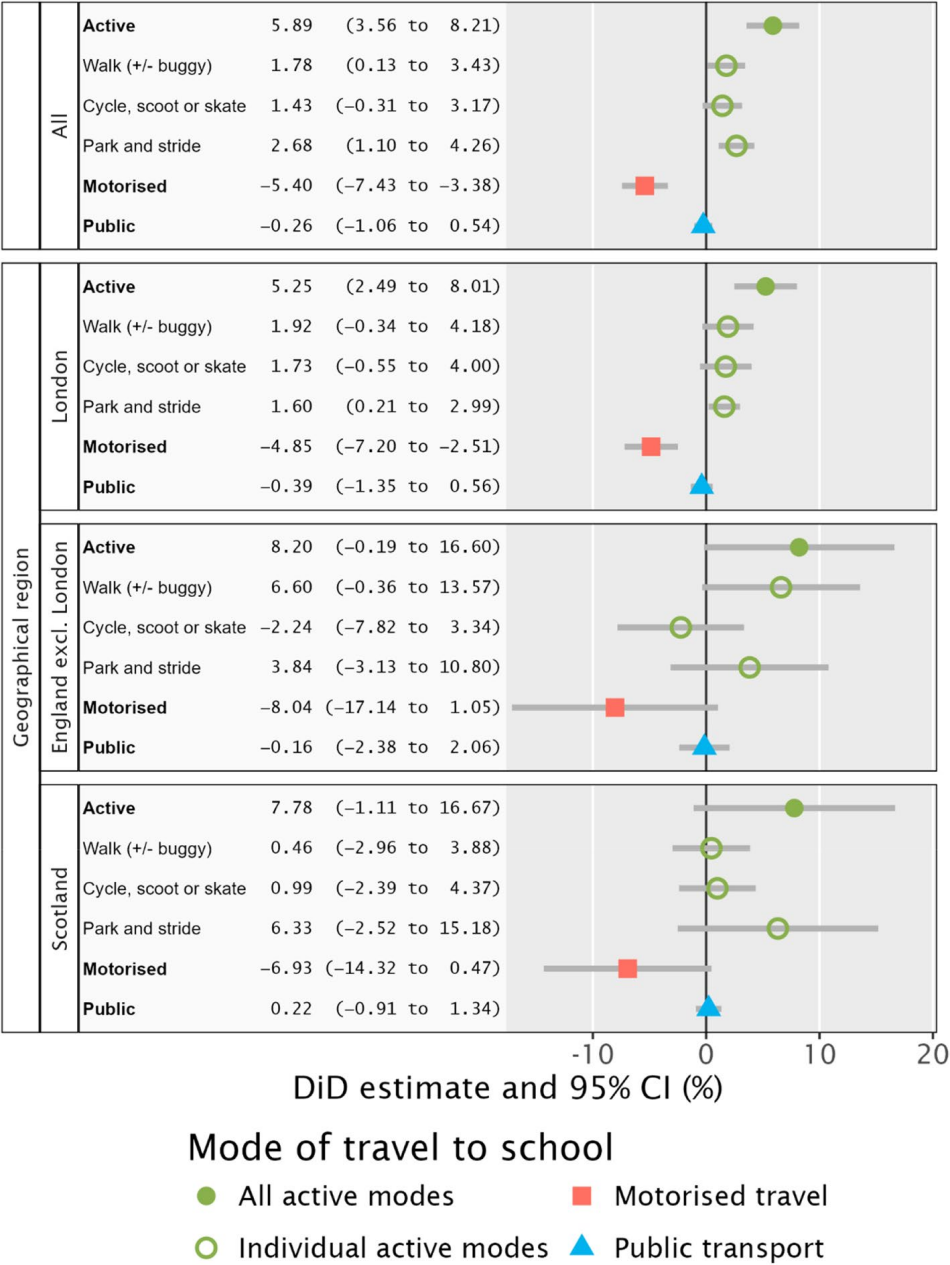
Changes in the percentage of pupils travelling by different modes in the total sample in intervention schools relative to controls, stratified by geographical region

**Fig. 3:**
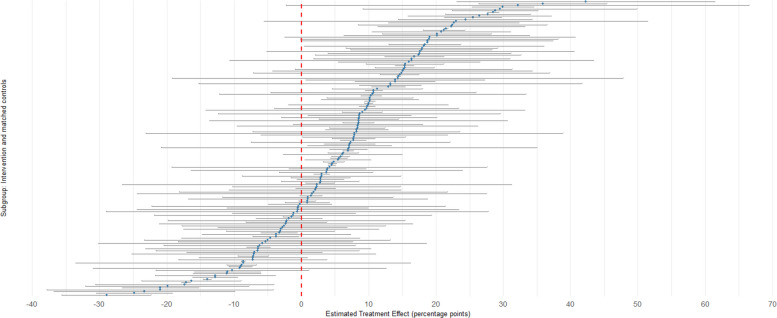
Caterpillar plot showing difference-in-difference estimate for each intervention school relative to its matched controls

Overall, the percentage of pupils travelling by active modes in intervention schools increased by 5.9% (95% CI 3.6 to 8.2) relative to control schools in absolute terms (Fig. 2) and by 8.4% (95% CI 5.1 to 11.7) in relative terms (Table 2). The largest contributor to the net increase in active travel was an increase in the percentage of pupils using park-and-stride. The percentage of pupils travelling by private motor vehicle decreased and no changes in the use of public transport or other modes of travel were seen. While the overall patterns of absolute and relative change were consistent, there was substantial variation in the size of these changes between schools (Fig. 3). In absolute terms, 122 of the 166 intervention schools saw an increase in active travel following the implementation of a traffic restriction scheme.

**Table 2:** Relative changes in the percentage of pupils travelling by different modes in total sample, stratified by geographical region

Country/region	Travel mode	Difference-in-difference estimate (95% CI)
Overall	Active transport	8.37 (5.07 to 11.67)
	Walk or buggy	3.38 (0.25 to 6.51)
	Cycle, scoot or skate	11.64 (−2.56 to 25.83)
	Park and stride	50.47 (20.71 to 80.23)
	Motorised travel	−24.79 (−34.08 to −15.50)
	Public transport	−3.49 (−14.29 to 7.32)
England, excl. London	Active transport	11.10 (−0.26 to 22.46)
	Walk or buggy	13.14 (−0.72 to 27.01)
	Cycle, scoot or skate	−14.90 (−52.07 to 22.27)
	Park and stride	44.27 (−36.13 to 124.66)
	Motorised transport	−35.12 (−74.82 to 4.57)
	Public transport	−5.01 (−75.10 to 65.08)
London	Active transport	7.38 (3.50 to 11.26)
	Walk or buggy	3.59 (−0.64 to 7.82)
	Cycle, scoot or skate	13.12 (−4.16 to 30.41)
	Park and stride	35.89 (4.75 to 67.04)
	Motorised	−24.54 (−36.40 to −12.67)
	Public transport	−4.33 (−14.82 to 6.17)
Scotland	Active transport	11.62 (−1.66 to 24.89)
	Walk or buggy	0.91 (−5.85 to 7.68)
	Cycle, scoot or skate	11.47 (−27.75 to 50.68)
	Park and stride	81.56 (−32.47 to 195.59)
	Motorised transport	−24.01 (−49.65 to 1.63)
	Public transport	11.45 (−48.33 to 71.22)

n represents number of intervention schools, each of which is matched to 2 controls

*CI* confidence interval

Results stratified by geographical region suggest that most of the overall effect was driven by results in London (75% of schools were in London); the pattern of results for England outside London and for Scotland was similar. Interaction terms were not statistically significant for area-level deprivation or method of enforcement (all p > 0.01), but for completeness the analyses stratified by these variables are shown in Supplementary Material, Table S6.

### Sensitivity analyses

Results from all three sensitivity analyses were consistent with the main results (Supplementary Material, Table S7). Matching on Modeshift STARS accreditation and using a one-year pre- and post-intervention window attenuated the size of the differences, but results that had been statistically significant in the main analysis remained so.

## Discussion

### Principal findings

This controlled natural experimental study evaluated the impact of traffic restriction schemes outside primary schools on children’s mode of travel to school in England and Scotland between 2012 and 2023. Following the schemes, the percentage of pupils using active modes of travel to school increased in both absolute and relative terms, with uptake of park-and-stride making the largest contribution to this increase. We also saw a reduction in the number of pupils making their entire journey in a private motor vehicle. There was no evidence for differences by geographical region, enforcement method or area-level deprivation of school location.

### Strengths and limitations

We identified, harmonised and combined the most complete datasets available on travel mode to school with covariates. This enabled us to include schools of different sizes, and with varying levels of deprivation and baseline active travel, in different geographical regions. We used measures of area-level deprivation based on school address rather than children’s home addresses, but state primary school catchment areas are comparatively small. We applied the difference-in-difference method using matched controls, comparing trends in the three years prior to intervention to give us confidence that control schools were well matched to intervention schools.

One limitation of the study is its reliance on data from hands-up surveys. These typically reflect answers given on a single day (and in this case only ask about travel to school), depend on children answering accurately and may be affected by social desirability bias, but the method has been shown to be valid and reliable [26]. The collection of these data is routine in Scotland –where travel mode to school is an official statistic – but is voluntary in England, which may have affected the quality of the data. If anything, measurement error in these data – if differential – would be expected to result in regression dilution and a bias of effect estimates towards the null, so it seems unlikely that the significant intervention effect we observed could be entirely explained by an artefact of data quality. In the absence of nationally representative data on individual children’s travel mode to school, we also had no information on children’s distance from home to school or mode of travel from school which is known to be an important influence on active travel [8] or the mode used home from school which may differ from mode used to school.

Although we controlled for a variety of observed differences between intervention and control schools in the analysis, we acknowledge that there may be unobserved reasons why some schools received the intervention and others did not, including the design of the local built environment, the strength of school leadership, other synergistic interventions, and the local political context. This raises the possibility of confounding by indication [34], whereby some of the schools receiving the intervention may have done so because they were already on an upward trajectory in the main outcome variable. The comparatively high baseline use of active modes in intervention schools is consistent with this explanation, but on the other hand our sensitivity analysis adjusting for school engagement in active travel promotion tends to contradict it. Another consideration for the generalisability of our findings is that our intervention schools were predominantly located in London. While the findings cannot be generalised to all schools, the intervention schools included in our analysis are comparable to all those that have implemented traffic restriction schemes, and can therefore be taken to reflect the contexts in which schemes have been implemented in practice.

### Interpretation

We found that implementing a traffic restriction scheme was associated with an average absolute increase in active travel of 5.9%, or an average relative increase of 8.4%, from an average baseline level of 70%. This equates to a Cohen’s D of 0.47, which is usually considered a moderate effect size [35] and is in line with the mean Cohen’s D of 0.5 from a previous meta-analysis of interventions to promote active transport to school [9]. Other studies into traffic restriction schemes outside schools are typically much smaller and uncontrolled, making it difficult to compare results. We are only aware of two controlled studies, one of which found an increase in active travel [36] and one a decrease in active travel [37]. An older review estimated that traffic restriction schemes reduce motor traffic on affected roads by between 30–64% [22]. A further uncontrolled study, looking at signage-only schemes, found minimal change in travel behaviour [24]. Our research is the only national, controlled evaluation of traffic restriction schemes and suggests that the disparity in previous findings may be due to small sample size or specific features of certain local areas or scheme designs.

Uptake of park-and-stride contributed most to the increase in active travel following scheme implementation. This suggests that in many cases families using a motor vehicle for the school run continued to do so, driving up to the boundary and completing the last part of the journey on foot, as previously reported in a study of park-and-stride in Oxfordshire, England [38] and in some drivers in a study of London’s Ultra-low Emission Zone [39]. Even short walking journeys like this may incorporate enough physical activity to benefit health [1], and they may also improve air quality around schools. At the same time there are concerns about the possibility of pollution, parking and related threats to pedestrian safety being displaced to boundary areas, which we were not able to examine in this analysis.

There was large heterogeneity in the success of these schemes between schools, which probably reflects aspects of the local social and physical context that we were not able to capture [40]. It is plausible that many schools with the most supportive contexts have already implemented traffic restriction schemes, at least within those local authorities that have embraced the principle. There is also some evidence to suggest that their implementation across London has been inequitable, with schemes tending to be implemented in less car-dominated environments and more polluted environments [41].

We found no evidence for a difference in effect by area-level deprivation, suggesting that any health benefits resulting from these schemes would accrue to children irrespective of the deprivation of the area in which they attended school. We found that these schemes have been disproportionately implemented in more deprived areas (Table 1), which tend to have poorer air quality, higher risk of road traffic injury and greater disease burden [42–44]. It is therefore plausible that these policies might contribute to reducing health inequalities.

### Policy implications

This study adds evidence in support of the potential active travel benefits of traffic restriction schemes. Restricting motor vehicle access to short sections of streets around schools for short periods of the school day is unlikely to result in large shifts in population travel behaviour on its own, but could form a necessary if not sufficient component of changing the wider transport system for children’s health and safety. Journeys to school contribute substantially to traffic volumes, for example accounting for a quarter of weekday morning peak car journeys in London [45]. Traffic restriction schemes might be combined with policies to reduce motor vehicle use among other groups and at other times of day, reducing overall emissions and increasing physical activity to improve both population and planetary health. While restricting motor vehicle use can generate objections or even conflict if some road users consider their freedoms to have been infringed [46], people tend to value local children’s health and safety and this may help local authorities make the case for policies aimed at reducing car use around schools.

### Future research

The heterogeneity in results between schools means that we cannot be certain about the likely effects of comparable investment in other schools in the future. Whilst these findings are promising, therefore, further monitoring and evaluation is warranted. Increasing investment to build research capacity within local government and foster collaborations between academic and local government has the potential to bridge the gap between research and policy and practice [47], for example by enabling local authorities to collect pre-intervention and control data that could be used to assess the effectiveness of future schemes.

Gaps remain in understanding how these schemes do or do not influence the travel modes used and total physical activity in children, and in quantifying their unintended consequences such as the displacement of traffic, congestion or air pollution. Ongoing causal loop diagramming and qualitative fieldwork in this study will enable us to map the potential systemic impacts of these schemes, elucidate the importance of the social and physical context of each school and help frame future evaluation studies [48].

## Conclusion

Implementing traffic restriction schemes around primary schools was associated with a net increase in active travel and a decrease in motor vehicle use for travel to and from school. Improving the availability, quality and consistency of data on school travel would facilitate future research into the health and planetary benefits of restricting motor vehicle traffic around schools.

## Supplementary Information

Supplementary Material 1.

## Data Availability

The datasets used in this study is managed by Walk Wheel Cycle Trust, TfL and Modeshift. Please apply to monitoring@sustrans.org.uk, travelforlife@tfl.gov.uk and admin@modeshift.org.uk.

## Abbreviations

UK: United Kingdom
IMD: Index of Multiple Deprivation
DiD: Difference-in-difference
CI: Confidence interval
