# Supplementary material for: Effectiveness and equity impacts of traffic restriction schemes outside schools: a controlled natural experimental study
Source: Int J Behav Nutr Phys Act. 2025 Dec 16;23:19. doi: 10.1186/s12966-025-01858-w (PMC12964863; doi:10.1186/s12966-025-01858-w)
Supplement: Supplementary file 1 — Supplementary Material 1. [file 12966_2025_1858_MOESM1_ESM.docx]

# Appendix

**Further details of cleaning for travel data**

For a subset of schools, we received data separately for each class. We aggregated these data to the school level for each available survey year for consistency with data from other schools, and conducted our analyses at the school level. In Scottish data, counts 1-4 were suppressed so to minimise the impact of this we excluded schools with more than 10% suppressed. This resulted in 29 schools being excluded from our analyses. Within Scotland, hands-up survey data is labelled according to school type (primary vs. secondary), and we used only primary school travel data. In England hands-up survey data is not labelled according to school type. We cross-referenced URNs with Department for Education 2020/21 School Census data for English schools and identified and removed state-maintained secondary schools.

**Appraisal of data sources**

**Table S1: Appraisal of possible data sources**

| **Data source** | **Coverage** | **Contains travel to school data** | **Contains school level data** | **Reason for exclusion** |
| --- | --- | --- | --- | --- |
| **Active Lives Children and Young People** | High (100,000+ responses per year) | Yes | No | Does not contain data about which school children attend |
| **The School Census** | High (all state schools in UK) | No | Yes | Does not contain measures of pupil travel behaviour |
| **The National Travel Survey** | Low (2,822-9,278 pupil-level responses per year) | Yes | Unknown | Even if school level data are available, insufficient coverage per school |
| **Hands-up survey data** | High (600–2900 schools per year from 2012-2024) | Yes | Yes | N/A |

**Table S2 Recoding travel modes for consistency**

| **Recoded value** | **Original values** | | |
| --- | --- | --- | --- |
|  | **Scotland** | **England non-London** | **London** |
| **Walk/buggy** | Walk | Walk | Walk  Buggy |
| **Cycle/scoot/skate** | Cycle  Scooting/skating | Cycle  Scooting/skating | Cycle  Scooting |
| **Park and stride** | Park and stride | Park and stride | Park and stride |
| **Private motor** | Driven  Taxi | Car  Car share  Motorbike/scooter | Car/motorcycle  Car share |
| **Public transport** | Bus | Public bus  School bus  Train/tube/metro | Public bus  School bus  Rail/overground  Tube  DLR  Tram  River |
| **Other** | Other |  |  |

**Further information on sources and classification of covariates**

We compared intervention school URNs in our datasets to those from Department of Education and corrected those that did not match the Register of Schools and Colleges in England (N=317). We also checked and harmonised local authority assignment. Where this changed during the study period as a result of changes in local authority boundaries, we retained the most recent classification [45]

Putative matching variables and covariates were nation/region (London, England non-London, Scotland, Wales), school size, urban/rural status, index of multiple deprivation (IMD) and baseline active travel. Nation/region was identified by the local authority and source of the data. The number of registered students (number on roll) was used as a measure of school size, and this was available from the Department for Education 2020/21 School Census data for English schools and the Summary statistics for schools in Scotland 2020 for Scottish schools.

The urban-rural status (urban/rural) of school location at the 2021 Census was determined by its Output Area (a small area used for calculating census statistics with a usual resident population of 100-625 people) [46]. Urban/rural status was recoded to a binary variable according to within-country standards. Index of Multiple Deprivation is an area-level composite deprivation score based on seven domains (income, employment, education, health, crime, barriers to housing and services, and living environment). A composite IMD designed to be comparable across Great Britain was matched based on school location using its Lower Super Output Area, which was the smallest geography for which we had this measure (usual population 1000-3000). IMD decile from 2019 was included as a continuous measure of deprivation, under the assumption of relative stability. We identified IMD by school site in English data using the postcode listed in School Census data. For Scottish schools, we downloaded 2023 spatial location data and used this to identify a school’s IMD [47]. Baseline active travel was the percentage of students travelling to school by active modes (walk/buggy, cycling/scooting/skating, and park and stride) in the pre-intervention year used for analysis.

**Table S3 Dichotomisation of urban/rural status**

| **England category** |  |
| --- | --- |
| A1: Major conurbation | Urban |
| B1: Minor conurbation | Urban |
| C1: City & town (not sparse) | Urban |
| C2: City & town (sparse setting) | Urban |
| D1: Town & fringe (not sparse) | Rural |
| D2: Town & fringe (sparse setting) | Rural |
| E1: Villages (not sparse) | Rural |
| E2: Villages (sparse setting) | Rural |
| F1: Hamlets and isolated dwellings (not sparse) | Rural |
| F2: Hamlets and isolated dwellings (sparse setting) | Rural |
| **Scotland category** |  |
| Large urban areas | Urban |
| Other urban areas | Urban |
| Accessible small towns | Urban |
| Remote small towns | Urban |
| Accessible rural | Rural |
| Remote rural | Rural |

**Overview of possible control methods**

**Figure S1: Comparison of Mahalanobis and Propensity Score Matching (PSM) at each ratio of control sites per intervention site
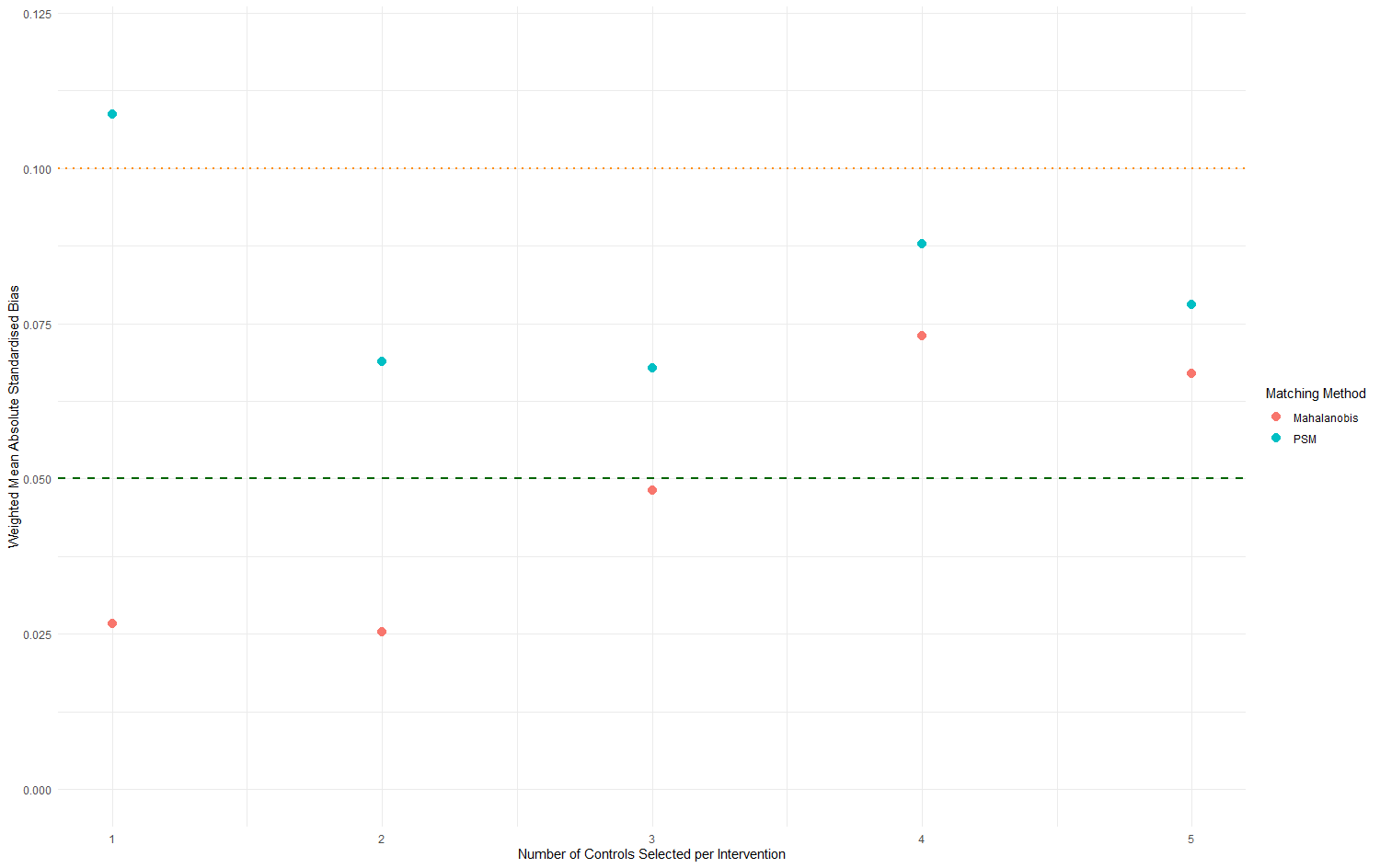
**

These mean absolute standardised biases are weighted to account for the number of schools per year. Mean absolute standardised bias was calculated separately for each intervention year at each ratio. This mean absolute standardised bias was multiplied by the proportion of schools with that intervention year to produce that year’s contribution to the overall weighted bias by ratio. Each year’s contribution to the standardised bias was then summed to create a weighted mean absolute standardised bias.

**Figure S2: Figure S2 Love plots showing the balance of covariates for each year of matching interventions to controls**

**
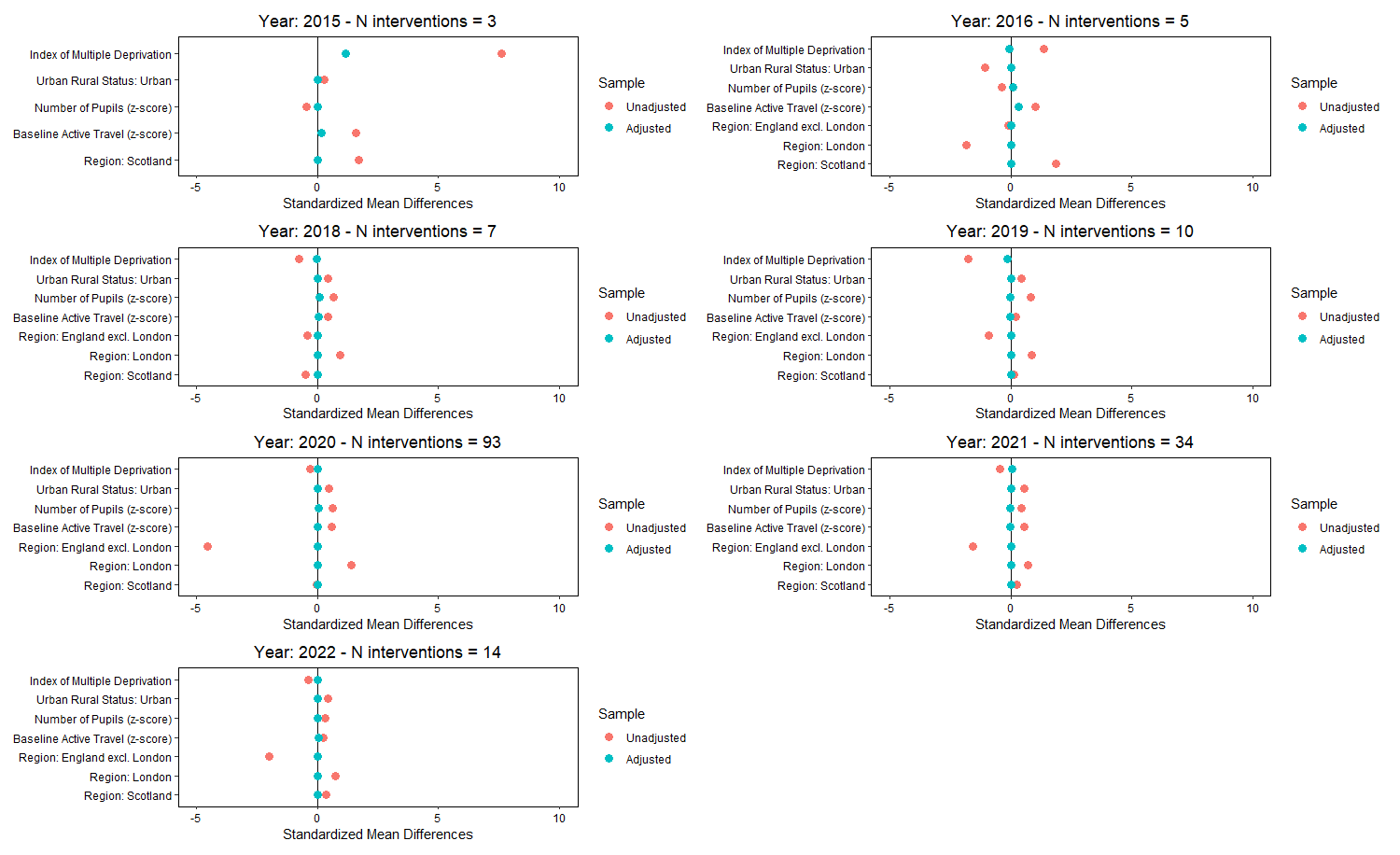
**

Note: Only covariates included for matching within a given year are included. In 2015, all intervention sites were in Scotland.

**Table S4: Summary of intervention schools by area and Local Authority**

| **Country** | **Local Authority** | **Number of intervention sites** |
| --- | --- | --- |
| England, excluding London | Birmingham City | 1 |
|  | Hampshire | 1 |
|  | Southampton City | 4 |
| London | Barnet | 2 |
|  | Brent | 11 |
|  | Bromley | 1 |
|  | Camden | 3 |
|  | Croydon | 2 |
|  | Ealing | 13 |
|  | Enfield | 7 |
|  | Greenwich | 3 |
|  | Hackney | 4 |
|  | Haringey | 8 |
|  | Harrow | 2 |
|  | Havering | 3 |
|  | Hounslow | 6 |
|  | Islington | 5 |
|  | Kingston upon Thames | 2 |
|  | Lambeth | 8 |
|  | Lewisham | 1 |
|  | Merton | 14 |
|  | Newham | 1 |
|  | Redbridge | 8 |
|  | Richmond upon Thames | 4 |
|  | Southwark | 3 |
|  | Sutton | 3 |
|  | Waltham Forest | 3 |
|  | Wandsworth | 7 |
|  | Westminster | 2 |
| Scotland | City of Edinburgh | 6 |
|  | East Ayrshire | 1 |
|  | Glasgow City | 25 |
|  | Perth and Kinross | 3 |

**Table S5: number of intervention sites per intervention year and the number of controls available to be matched**

| **Intervention year** | **Number of intervention sites** | **Number of available controls** |
| --- | --- | --- |
| 2015 | 3 | 502 |
| 2016 | 5 | 461 |
| 2017 | 0 | - |
| 2018 | 7 | 591 |
| 2019 | 10 | 509 |
| 2020 | 93 | 735 |
| 2021 | 34 | 892 |
| 2022 | 14 | 943 |

**Table S6: Stratified results for differences by area level deprivation and method of enforcement**

|  | **DiD estimate (95%CI)** | | |
| --- | --- | --- | --- |
| **IMD quintile** | **Active travel (%)** | **Private motor (%)** | **Public transport (%)** |
| 1 - Highest deprivation (n = 38) | 4.15 (-1.74 to 10.05) | -3.34 (-8.14 to 1.46) | -0.59 (-2.35 to 1.18) |
| 2 (n = 44) | 5.30 (1.36 to 9.24) | -4.71 (-7.88 to -1.54) | -0.35 (-1.82 to 1.12) |
| 3 (n = 40) | 5.63 (1.56 to 9.71) | -5.67 (-9.18 to -2.17) | 0.19 (-1.15 to 1.53) |
| 4 (n = 18) | 5.58 (-1.93 to 13.08) | -5.66 (-12.03 to 0.70) | 0.54 (-1.29 to 2.38) |
| 5 - Lowest deprivation (n = 26) | 10.02 (2.97 to 17.06) | -8.99 (-15.46 to -2.53) | -0.86 (-2.12 to 0.41) |
| **Method of enforcement** |  |  |  |
| ANPR-enforced (n = 99) | 5.68 (2.33 to 9.02) | -5.42 (-8.03 to -2.80) | -0.26 (-1.49 to 0.97) |
| Not ANPR-enforced (n = 67) | 6.20 (2.45 to 9.95) | -5.38 (-8.61 to -2.15) | -0.26 (-1.35 to 0.84) |

Note: n represents number of intervention sites, each of which is matched to 2 controls.

**Table S7: Results from sensitivity analyses for relative differences in the percentage of pupils travelling by different modes in total sample, stratified by nation/region**

|  |  | **DiD estimate (95% CI)** | | |
| --- | --- | --- | --- | --- |
|  | **n** | **Active travel (%)** | **Private motor (%)** | **Public transport (%)** |
| **Fractional logit model (% change)*** | 166 | 6.33 (4.06 to 8.46) | -5.61 (-7.29 to -3.77) | -0.37 (-1.15 to 0.51) |
| **Matching on STARS accreditation** | 129 | 5.40 (2.60 to 8.20) | -4.98 (-7.42 to -2.53) | -0.42 (-1.29 to 0.45) |
| **1 year pre- and post-intervention** | 157 | 5.77 (3.61 to 7.93) | -4.64 (-6.55 to -2.74) | -1.10 (-1.76 to -0.44) |

*Calculated from coefficients using the following formula:

Baseline log odds = log(baseline / (1 – baseline))

New log odds = baseline log odds + coefficient

Predicted = exp(new log odds) / (1 + exp(new log odds))

Change = (Predicted – baseline) *100

For example, active travel coefficient = 0.32, baseline active travel = 0.70

Baseline log odds = log(0.70/0.3) ~ 0.85

New log odds = 0.85 + 0.32 ~ 1.18

Predicted = exp(1.18)/ 1+exp(1.18) ~ 0.76

Change = 0.76-0.70 ~ 0.633 ~ 6.33
